# A model for cyclic mechanical reinforcement

**DOI:** 10.1038/srep35954

**Published:** 2016-10-27

**Authors:** Zhenhai Li, Fang Kong, Cheng Zhu

**Affiliations:** 1 Wallace H. Coulter Department of Biomedical Engineering, Georgia Institute of Technology, Atlanta, GA 30332, USA; 2 Parker H. Petit Institute for Bioengineering and Bioscience, Georgia Institute of Technology, Atlanta, GA 30332, USA; 3 Molecular Modeling and Simulation Group, National Institutes for Quantum and Radiological Science and Technology, 8-1-7 Umemidai, Kizugawa, Kyoto 619-0215, Japan; 4 Singapore MIT Alliance of Research and Technology, Infectious Disease IRG, Singapore 138602, Singapore; 5 Woodruff School of Mechanical Engineering, Georgia Institute of Technology, Atlanta, GA 30332, USA

## Abstract

Mechanical force regulates a broad range of molecular interactions in biology. Three types of counterintuitive mechanical regulation of receptor–ligand dissociation have been described. Catch bonds are strengthened by constant forces, as opposed to slip bonds that are weakened by constant forces. The phenomenon that bonds become stronger with prior application of cyclic forces is termed cyclic mechanical reinforcement (CMR). Slip and catch bonds have respectively been explained by two-state models. However, they assume fast equilibration between internal states and hence are inadequate for CMR. Here we propose a three-state model for CMR where both loading and unloading regulate the transition of bonds among the short-lived, intermediate, and long-lived state. Cyclic forces favor bonds in the long-lived state, hence greatly prolonging their lifetimes. The three-state model explains the force history effect and agrees with the experimental CMR effect of integrin α_5_β_1_–fibronectin interaction. This model helps decipher the distinctive ways by which molecular bonds are mechanically strengthened: catch bonds by constant forces and CMR by cyclic forces. The different types of mechanical regulation may enable the cell to fine tune its mechanotransduction via membrane receptors.

Mechanical forces are ubiquitous inside and outside of cells^1^,^2^,^3^. Cells sense, generate and exert forces. Cell behaviors and fates can be regulated by force, e.g., via influencing interactions among molecules that generate and/or support forces. Among these molecules, cell adhesion molecules (CAMs) are of particular interest as they provide specific molecular bridges across the cell membrane. Inside the cell, CAMs are usually linked to cytoskeleton either directly or through adaptor proteins; whereas outside the cell, CAMs form noncovalent bonds with ligands on other cells or on the extracellular matrix (ECM), forming an integrated structure to bear force.

The stability of bonds between CAMs and ligands is usually regulated by mechanical force. Several types of mechanical regulation have been observed under constant forces, in which a single ramp to a clamped force was applied to the molecular bond to regulate its dissociation (force-clamp spectroscopy, Fig. 1a,b). Intuitively, mechanical force acting on molecular bonds should accelerate dissociation by shortening their lifetimes, termed slip bond (Fig. 1b, red). This was originally proposed by Bell^4^ and has been observed in many molecular systems^5^. The opposite behavior has been proposed based on theoretical considerations, such that force may also slow bond dissociation by prolonging their lifetimes, a counterintuitive behavior termed catch bond^6^ (Fig. 1b, black). Molecular bonds whose dissociation is independent of force were termed ideal bond (Fig. 1b, blue), which have also been recently observed^7^. Unlike the prevalent slip bonds, catch bonds are only observed in a range of forces, beyond which they transition to slip bonds^8^,^9^,^10^,^11^,^12^,^13^,^14^ (Fig. 1b, black). Since the experimental demonstration of catch-slip bonds, several theoretical models have been proposed based on physical considerations and structural observations^15^,^16^,^17^,^18^,^19^,^20^,^21^,^22^,^23^,^24^. Some suggest force inducing allosteric changes to strengthen the bonds^19^,^20^,^21^,^22^,^23^. Others propose two states and/or two dissociation pathways with different dissociation rates, one short-lived and fast-dissociating whereas the other long-lived and slow-dissociating. In a two-state model, force applied on the bond switches the bond state from short- to long-lived, thereby prolonging the average lifetime^15^,^16^,^17^,^18^,^24^,^25^.

More recently, a new type of mechanical regulation has been observed for the interactions of two integrins, α_5_β_1_ and α_L_β_2_, with their respective ligands, fibronectin (FN) and intercellular adhesion molecule 1. This phenomenon, termed cyclic mechanical reinforcement (CMR)^26^, refers to the observation that a prior cyclic force on a bond prolongs its lifetime by orders of magnitude without changing the clamped force at which the bond lifetime is subsequently measured. Two different cyclic force waveforms were applied to the bond^26^: 1) a single loading-unloading cycle before clamping the force constant (single-cycled CMR, Fig. 1c), and 2) multiple loading-unloading cycles before clamping the force constant (multi-cycled CMR, Fig. 1e). The extent of bond lifetime prolongation increases with the amplitude of the cyclic force of single-cycled CMR (Fig. 1d) and the number of force cycles of multi-cycled CMR (Fig. 1f). This suggests that, in addition to the present level of force, the history of force application is memorized by the bond that later alters its lifetime. Other manifestations of force history effect have been observed and discussed in other systems^10^,^17^,^27^,^28^. However, most two-state catch bond models assume average lifetime as a single-valued function of force at the time when lifetime is measured without considering the prior history of force, which is inadequate for the CMR phenomenon. Recently, a two-state model was examined for the ability to account for force history effects^29^. This model was able to generate single-cycled CMR effect but the multi-cycled CMR was attributed to the pre-matured dissociation of short-lived bonds during loading-unloading cycles prior to lifetime measurement. However, this possibility was specifically ruled out experimentally in the original CMR study^26^. In this model, bond strengthening by CMR is not distinct from that by catch bonds, yet the biophysical mechanisms underlying these two phenomena may be conceptually different.

In this paper we describe a model for CMR that assumes bond dissociation from three states governed by the Bell model, with state occupancies regulated by force. The validity of the three-state model has been supported by its ability to fit several types of force regulation of molecular dissociation, including CMR, slip bond, and catch-slip bond. Importantly, in this model catch bond and CMR strengthen molecular interaction through different pathways, consistent with the proposal that catch bonds and CMR are based on distinct structural mechanisms^26^. This enables us to predict the circumstances under which a molecular interaction exhibits catch bond, slip bond, or CMR.

## Results

### Conceptualization and energy landscape

We constructed our model by conceptualizing an energy landscape. In previous two-state catch-slip bond models, molecular bonds may reside in one of two stable states and dissociate from either^16^,^30^. The two stable states are represented by two energy wells whose relative depths determine relative bond occupancies in the two states. Mechanical force tilts the energy landscape, alters the energy well depths, and shifts the bond occupancies from the short-lived to long-lived state to give rise to a catch bond. Our model adds an intermediate state that either connects or segregates the short- and long-lived states depending on force, generating two bidirectional transition pathways to link the three states. One pathway allows transition between the short-lived and intermediate states and the other pathway allows transition between the intermediate and long-lived states, without direct transition between the short- and long-lived states. Increasing force promotes transition from the short-lived state to the intermediate state but suppresses transitions in the other three directions. Decreasing force resumes all transitions and enables bond occupancies accumulated in the intermediate state during the loading phase to transition to the long-lived state. Therefore, the intermediate state stores bonds coming from the short-lived state during loading and dispatches them to the long-lived state during unloading.

**Figure 2:**
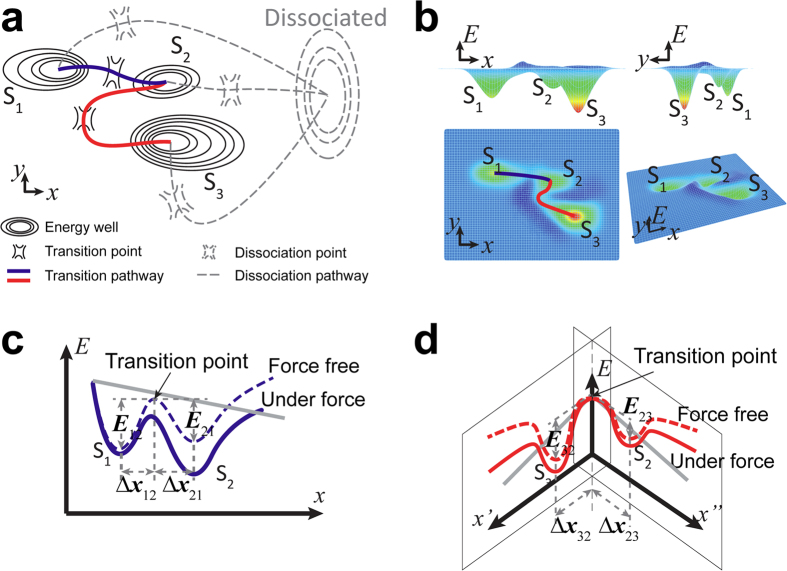
Energy landscape. (**a**) A simplified contour plot of the energy landscape of the three-state model illustrating three bound states, one dissociated state, and the pathways connecting these states. The *x-y* plane represents the space spanned by the reaction coordinates. Force is along the *x*-axis direction. (**b**) The 3-D energy landscape of state transitions excluding the dissociated state and dissociation pathways. The *x-y* plane is the same as in (**a**). The *z*-axis represents energy *E*. The color-coded height indicates the free energy level. (**c**) The energy landscape along the transition pathway between the short-lived state and the intermediate state. (**d**) The energy landscape along the transition pathway between the intermediate state and the long-lived state.

An energy landscape that displays the aforementioned properties can be constructed (Fig. 2a). The *x-y* plane maps to the space spanned by the reaction coordinates. The *x*-axis is along the direction of force. The *y*-axis is perpendicular to force. Three energy wells depicted by solid contours represent the three stable bound states, and one energy well depicted by dashed contours represents the dissociated state. The two transition pathways connecting the short-lived state to the intermediate state and then to the long-lived state are illustrated by the solid curves. The three dissociation pathways connecting the three bound states to the dissociated state are illustrated by the dashed curves. For clarity, we hid the dissociated state and dissociation pathways in Fig. 2a, and plotted the energy landscape for state transition only in a 3-D view with the vertical axis representing free energy *E* (Fig. 2b). The depths of energy wells are indicated by color coding. The heights of the energy barriers determine the force-free transition rates between the neighboring states. The two transition pathways connecting the three states are highlighted by the blue and red curves in the top view.

To illustrate how force tilts the energy landscape, we split the energy landscape based on the two pathways colored in blue and red, and project them onto the *x-E* plane (Fig. 2c) or look into the origin along the diagonal line *x* = y = *E* (Fig. 2d). The dashed curves in Fig. 2c,d show the force-free energy landscape. The solid curves depict the energy landscape tilted by force. Force is applied along the direction of the transition pathway from the short-lived state to the intermediate state. Therefore, it shallows the energy well of the short-lived state and deepens the energy wells of both the intermediate and long-lived states, promoting transition from the short-lived state to the intermediate state and suppressing transitions in all three other directions. Releasing force restores the energy landscape, resuming transitions between the intermediate state and the long-lived state as well as from the intermediate state to the short-lived state. The energy barrier from the intermediate state to the long-lived state is lower than that from the intermediate state to the short-lived state such that more bond occupancies from the intermediate state are transitioned to the long-lived state than the short-lived state as force is released.

### Master equations

To describe the energy landscape mathematically, we set up a set of master equations. In our model, the likelihood of total bond survival over time obeys first-order dissociation, 
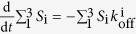
, where *S*_i_ and 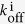 denote the occupancy of *i*th state and the off-rate of bond dissociation from the *i*th state (*i* = 1, 2, and 3 denoting the short-lived, intermediate, and long-lived state, respectively). The inner exchange of bond occupancies also obeys first-order kinetics:


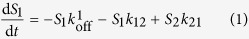



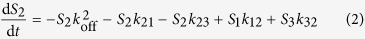



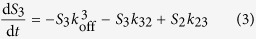


The first term on the right-hand side of each equation accounts for dissociation at rates of 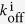 (*i, j* = 1–3), whereas the other terms account for the inner-exchange of bond occupancies at rates of *k*_ij_ (*i, j* = 1–3). The transition rates are assumed to follow the Bell equation^4^: 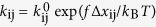, where 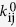 is the force-free transition rate, *f* is force, Δ*x*_ij_ represents the distance from the bottom of the *i*th energy well to the point of transition to the *j*th energy well, *k*_B_ is Boltzmann constant, and *T* is absolute temperature. Dissociation rates are also assumed to follow the Bell equation: 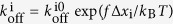, where the parameters are of similar definitions as the transition rates. The average lifetime can be calculated by: 
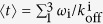
, where 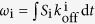 represents the fraction of bonds dissociated from the *i*th state and satisfies^31^: 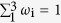.

### Comparison to integrin CMR experiment

**Figure 3:**
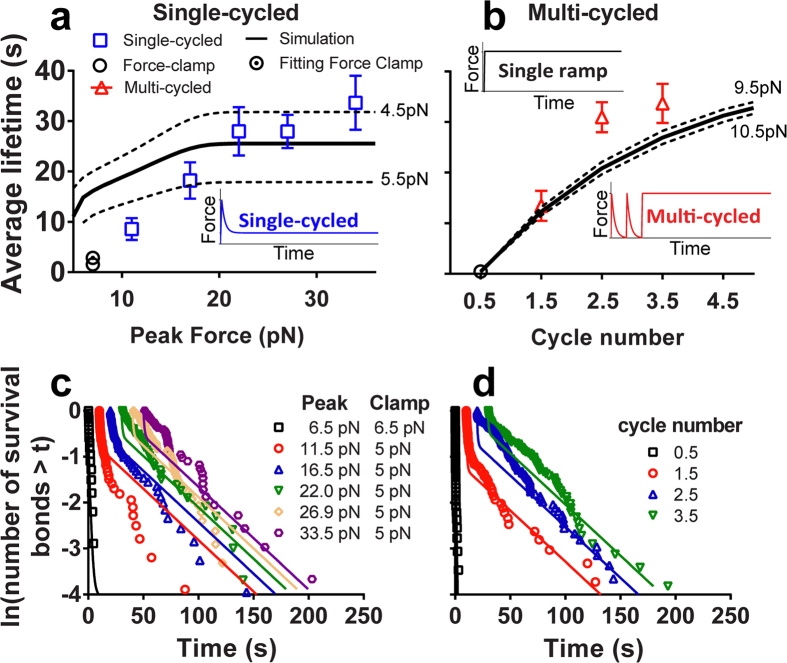
Comparison of measured and predicted bond lifetimes. (**a**) Single-cycled CMR with a range of peak forces followed by lifetime measurement at 5-pN clamped force, and force-clamp measurement at 6.5-pN. (**b**) Multi-cycled CMR with a range of cycle numbers followed by lifetime measurement at 10-pN clamped force. Inserts show the respective force histories prior to bond lifetime measurement at the clamped force. (**c,d**) Lifetime distributions of single-cycled (**c**) and multi-cycled (**d**) CMRs. The chi squares of the fitting to single-cycled and multi-cycled distributions are 130.2 and 208.1 respectively. The symbols are experimental data from Ref. 26 with different shapes and colors representing indicated experiment conditions. Solid curves are the best-fit solutions of the three-state model. Dashed curves in (**a**,**b**) are model predictions with the indicated force variations.

Previous experiments demonstrating the CMR phenomenon were performed with three different loading waveforms^26^: 1) force-clamp, 2) single-cycled CMR, and 3) multi-cycled CMR (Fig. 3a,b insets). While force-clamp elicits catch bonds^11^, both single-cycled and multi-cycled CMRs further prolong bond lifetime by orders of magnitude^26^. Bond lifetimes increase with increasing peak force in single-cycled CMR (symbols in Fig. 3a) and increasing cycle number in multi-cycled CMR (symbols in Fig. 3b), before reaching a plateau. The lifetimes distribute multi-exponentially (Fig. 3c,d), suggesting that bonds dissociate from multiple states along multiple pathways. Lifetime prolongation is due to bond occupancies shifting from the short-lived state to the long-lived state (symbols in Fig. 3c,d and Supplementary Fig. S1)^26^.

To compare with data, we assumed that all bonds occupy the short-lived state initially and are subjected to specific force cycles (see Methods). By integrating the master equations over time along the given force history, we obtained the fraction of bonds dissociated from each state (*ω*_i_), and average time (<*t*>) of total bond survival, i.e., bond lifetime as measured from experiment (Fig. 3, Supplementary Fig. S1, and Supplementary Information). Using Monte Carlo Least-Squares fitting^32^, we obtained the best-fit parameters by comparing the model solutions with two sets of data, one from single-cycled (Fig. 3a,c) and the other from multi-cycled (Fig. 3b,d) CMR experiments. Each set contains multiple experiments with a range of peak forces or cycle numbers. Data from each experiment are plotted as a distribution of bond lifetimes and their average (Fig. 3).

Supplementary Table S1 lists the best-fit parameters. 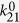 and 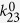 are significantly faster than 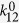 and 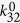, suggesting that the intermediate state is the least stable in the absence of force. 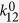 is the slowest, indicating short-lived state is the most stable state without force. Fitting returned one positive (1 → 2) and three negative (2 → 1, 2 → 3, and 3 → 2) Δ*x*_ij_ values for inner-exchanges of states (Supplementary Table S1), indicating that one of these transition directions is along and the others are opposite to the force direction, consistent with the conceptual energy landscape. The absolute values of Δ*x*_ij_ vary from 1.35–6.80 nm, indicating that the inner-exchanges of states are highly sensitive to force. The best-fit Δ*x*_i_ values are all positive. The Δ*x*_i_ for dissociation from the short-lived and intermediate states are 1.08 and 1.31 nm, respectively, again indicating the high force sensitivity of the dissociation rates. The high force sensitivity of these rates may be understood in terms of force-induced integrin conformational changes, which may propagate to the ligand binding site to regulate dissociation kinetics. Integrin is a large protein, with a ~10 and ~20 nm linear dimension in the bent and extended conformations^33^ that can change back and forth reversibly under force^34^,^35^. Such conformational changes could have a leverage effect, resulting in higher force sensitivity of rate coefficients, manifested as nanometer range transition and dissociation distances. It is worth noting that the Δ*x* of dissociation from the long-lived state is 0.03 nm, indicating that the dissociation pathway is nearly perpendicular to the force direction. Further analysis of transition directions confirmed that these directions are the most appropriate combination for CMR (Supplementary Fig. S2). The solutions compare well with bond lifetime distributions from both single-cycled (Fig. 3c) and multi-cycled (Fig. 3d) CMR experiments. The predicted average bond lifetimes also follow the changing trends of the data with the increasing amplitude (Fig. 3a) and number (Fig. 3b) of force cycles. In addition, the simulated fraction of bonds dissociated from each state follows similar trend of that estimated from experimental data (Supplementary Information and Fig. S1).

### Parametric analysis

Parametric analysis reveals that the multi-cycled CMR solution is quite robust, as ± 0.5-pN change around the 10-pN clamped force generates nearly indistinguishable average post-cycle bond lifetime curves (Fig. 3b). By comparison, the single-cycled CMR solutions are very sensitive to the clamped force, as a ± 0.5-pN change around the 5 pN value results in a ~10-s variation in the average post-cycle bond lifetime curve (Fig. 3a). Considering the experimental difficulties to precisely control the clamped force and loading/unloading patterns at low forces and the fact no freely adjustable parameter is used in this comparison, our model does a good job to predict the data trends for both single-cycled and multi-cycled CMR experiments. The modest deviation between model solution and the low peak force data from the single-cycled CMR experiments is considered acceptable.

Thorough parametric analysis was carried out to show how 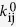, Δ*x*_ij_, and loading/unloading rates influence the model predictions (Supplementary Information and Figs S3 and S4). Interestingly, the slower the loading or unloading, the longer the bond lasts. The frequency of the cyclic force is determined by both loading and unloading rates. Slower loading/unloading rates lead to lower cyclic frequency. It has been reported that cells in focal adhesions generate ~0.1 Hz dynamic forces^36^, much lower than the ~0.5 Hz cyclic force in our previous multi-cycled CMR experiment^26^, suggesting that cells may use cyclic forces to strengthen their focal adhesions to the ECM (see Supplementary Information).

We assumed that initially the integrin resides in the short-lived state because crystallographic and EM studies suggest that the bent conformation is more favorable for integrins in the absence of ligand. To evaluate the effects of this assumption, we examined the dependence of the average bond lifetime on the initial condition. As the initial bonds shifted from the short-lived state to the long-lived state, the calculated average lifetimes increase in both single-cycled and multi-cycled loading cases, but the CMR effects become less and less pronounced (Supplementary Fig. S5). The reduced CMR effect is due to the fact that bonds initially in the long-lived state have already assumed the longest lifetimes even without cyclic force exerting on them.

### Effects of changes in state occupancies

**Figure 4:**
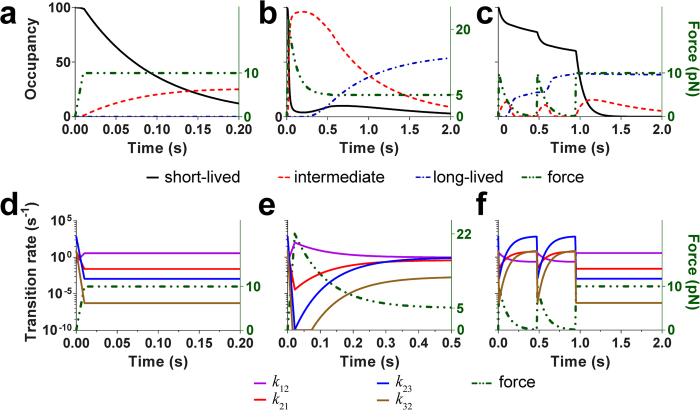
Occupancy and inner state transition rate variations along different force histories. (**a–c**) Force (green, right ordinate) and occupancy (left ordinate) curves of the three states over time for force clamp (**a**), single-cycled CMR (**b**), and multi-cycled CMR (**c**). (**d–f**) Force (green, right ordinate) and rate (left ordinate) curves of four transitions over time for force clamp (**d**), single-cycled CMR (**e**), multi-cycled CMR (**f**).

In experiment and model, bond lifetime is defined from the moment when the force reaches the clamped level to the moment of bond dissociation. A prior force history exerts its effect by changing the state occupancies of bonds at the “initial” time from which their lifetimes are measured. To investigate how a prior force history affects the initial occupancies, we tracked real time transition rates and real time state occupancies during loading-unloading along three specific force histories: a single ramp to 10-pN clamped force, 2.5-cycles of loading-unloading between 0 and 10 pN followed by clamping at 10 pN, and a single-cycle of loading-unloading with 22-pN peak force and 5-pN clamped force. By integrating the master equations, the transition rates and state occupancies were obtained in these prior force histories (Fig. 4).

As shown in Fig. 4a–c, for all three prior force histories, loading and unloading cycle(s) promote bond redistribution among the three states, using the intermediate state as a relay station. In the force clamp phase, the inner exchange between short-lived and intermediate state reaches equilibrium and that between intermediate and long-lived state is suppressed. Therefore, bonds dissociate from the states that they occupied at the time without transitioning to other states. The fraction of bonds dissociated from the intermediate state is low because of its low stability (cf. its shallow energy well in Fig. 2). Because of its two orders of magnitude faster transition rate to the long-lived than to the short-lived state at low or zero forces (Fig. 4e,f), most bonds in the intermediate state transition to long-lived state during unloading prior to force clamping.

### Similarities and differences among slip bond, catch-slip bond, and CMR

**Figure 5:**
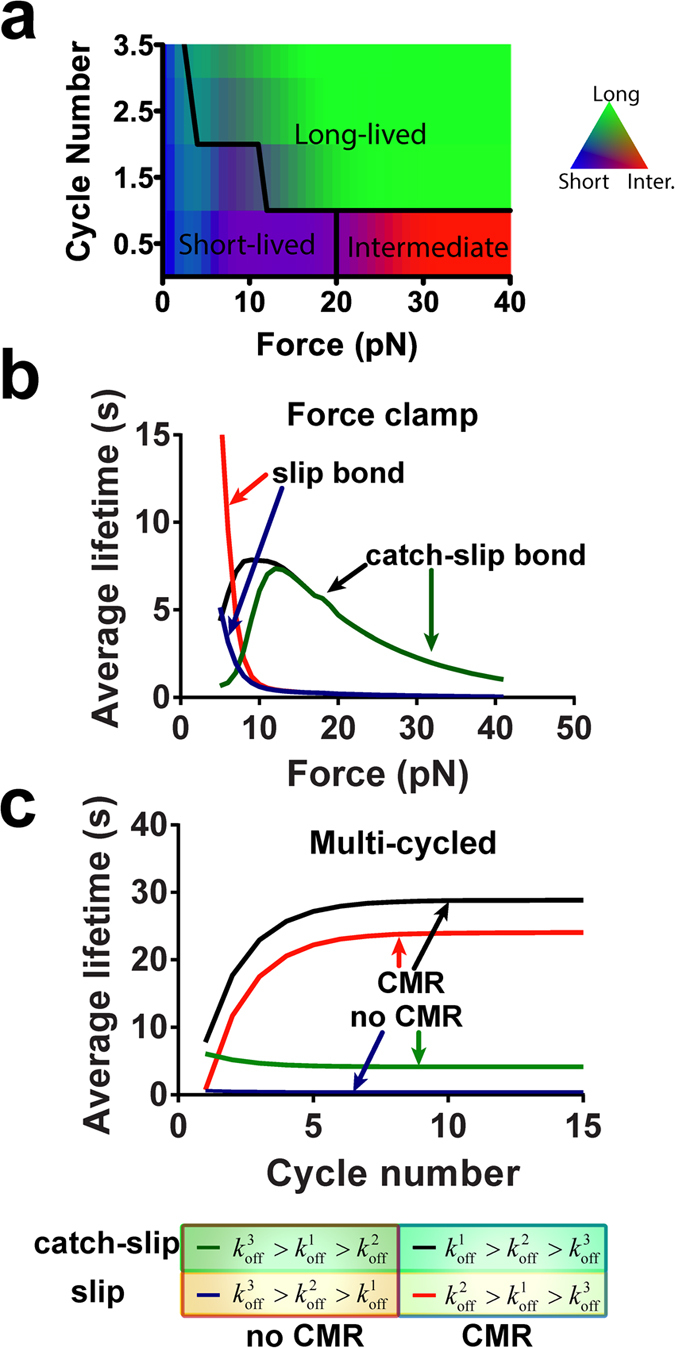
Phase diagram and model. (**a**) Force vs. cycle number phase diagram. (**b,c**) Predicted lifetime with different off-rates of three states under single ramp (**b**) or multi-cycled forces (**c**). Parameters used in (**a**) are those best-fit the integrin α_5_β_1_–FN CMR data in ref. 26. Parameters used in (**b**,**c**) are listed in Supplementary Table S2.

To further investigate how prior force histories affect the state occupancies, we generated a phase diagram by simulating multi-cycled CMR over a force vs. cycle number space ranging from 1–40 pN and 0.5–3.5 cycles using the parameter set that best-fits the integrin α_5_β_1_–FN CMR data. Each point in this force-cycle number plane is color-coded with RGB values according to the fractions of bonds dissociated from the three states. This phase space was then divided into three regions according to the dominant state from which most bonds dissociate (Fig. 5a). At low forces and low cycle numbers, most bonds dissociate from the short-lived state. At high forces with a single force ramp, most bonds dissociate from the intermediate state. In the remaining region, most bonds dissociate from the long-lived state. This phase diagram reveals differential effects of amplitude and cycle number of dynamic forces on the state occupancy of bonds, which is a key mechanism for CMR. It also suggests different biophysical mechanisms for catch bonds and CMR: A constant force with a single ramp promotes bonds transition to the intermediate state to give rise to catch bonds, whereas cyclic forces promote bond transition to the long-lived state to generate CMR effects.

The distinctive biophysical mechanisms for catch bonds and CMR afford the possibility of different types of mechanical regulation of molecular bonds by constant vs. cyclic forces. For instance, if 
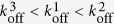
, increasing levels of constant force would drive bond occupancy to the intermediate state with accelerated dissociation to produce a slip bond. In comparison, increasing the number of loading-unloading cycles would drive bond occupancy to the long-lived state with decelerated dissociation to exhibit CMR. To illustrate this, we ran four sets of single ramp force (Fig. 5b) or multi-cycled cyclic force (Fig. 5c) simulations with different off-rates (Supplementary Table S2). As predicted, 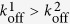 resulted in catch-slip bonds, and 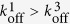 resulted in CMR.

**Figure 6:**
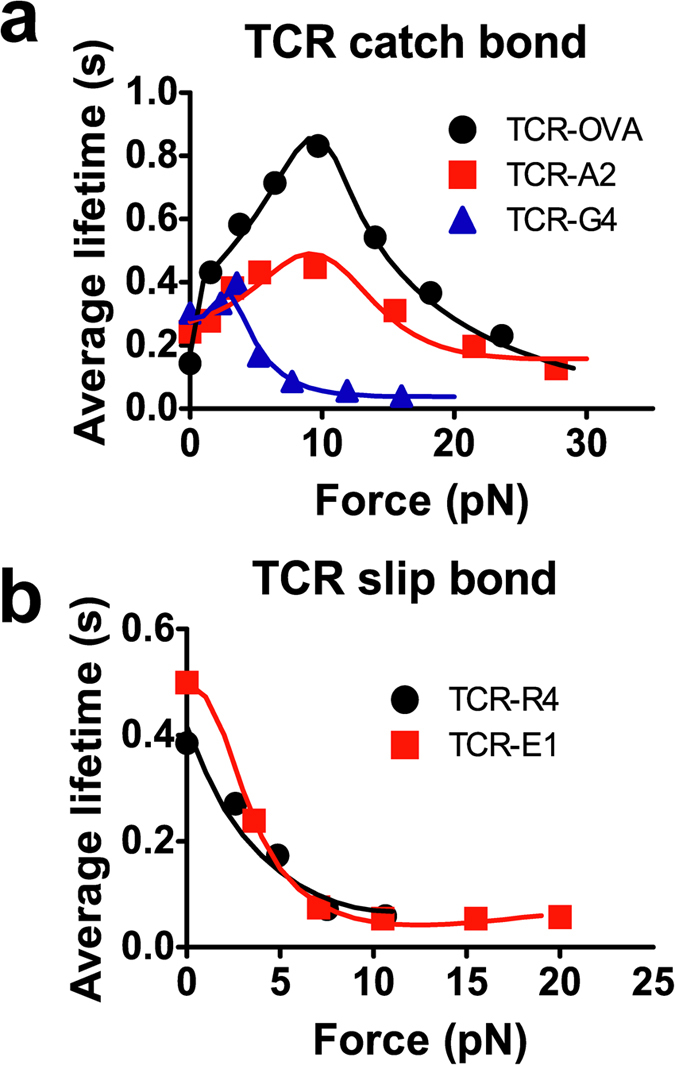
Fits to catch and slip bonds. Comparison between measured and predicted bond lifetime of TCR–pMHC interactions that exhibit catch-slip (**a**) and slip-only (**b**) bonds. The symbols are experimental data from ref. 13. The curves are best-fit solutions of the model.

The ability of the present model to describe catch and slip bonds was further tested against the data of T-cell receptors (TCRs) interacting with peptides bound to major histocompatibility complex (pMHC) molecules^13^,^37^,^38^,^39^. Our model fits the bond lifetime vs. clamped force data well for both peptides of high biological activities, which behave as catch-slip bonds, and peptides of low biological activities, which behave as slip-only bonds^13^ (Fig. 6, see Supplementary Table S3 the best-fit parameters). For all cases, the transition rates from the intermediate state to the long-lived state are close to 0, indicating that very few bonds transition to the long-lived state, essentially reducing the model from three-state to two-state. For the slip-only bonds, the transition rates from the short-lived state to the intermediate state are nearly 0, indicating that very few bonds transition to the intermediate state, which essentially reduces our model to the Bell model^4^. The reductions are expected because simpler data require simpler models.

## Discussion

### Possible source of fitting errors

In some cases our model fits deviate from the experimental lifetime distributions, especially for the short lifetimes in single-cycled CMR with larger peak forces and in multi-cycled CMR with higher cycle numbers (Fig. 3c,d). Possible causes for such discrepancies may include: 1) Short lifetime distributions are determined by fast-dissociating bonds from the short-lived and intermediate states. The fractions of bonds dissociated from the intermediate state are low in all cases (Supplementary Fig. S1), preventing us from obtaining a reliable fitting value of the off-rate for the intermediate state. 2) All initial bonds were assumed to reside in the short-lived state, which may result in more short-lived bonds dissociated from that state. 3) Bonds dissociation during loading-unloading was constrained to avoid pre-matured bond dissociation. Since the short-lived bonds dissociated the fastest, this constraint may result in higher fraction of bond dissociated from the short-lived state after force arrived at the clamped level for bond lifetime measurement. 4) Since the unloading is a non-linear process, finding the precise endpoint of the unloading in experiment is difficult, making the starting point of the lifetime inaccurate, which preferentially impacts the accuracy of the short lifetime measurements.

Since decreasing the initial occupancy of short-lived bond would reduce the CMR effect (Supplementary Fig. S5), we only tested if removing the constraint of dissociation during loading-unloading could improve the fitting. Reduced chi squares indicate that a better fitted lifetime distribution could indeed be obtained, returning a new best-fit Δ*x*_12_ value of 0.76 nm (Fig. 3 and Supplementary Fig. S6). However, to avoid the influence of pre-matured bond dissociation, we chose to constrain the dissociation during loading-unloading.

### Physical analogies

**Figure 7:**
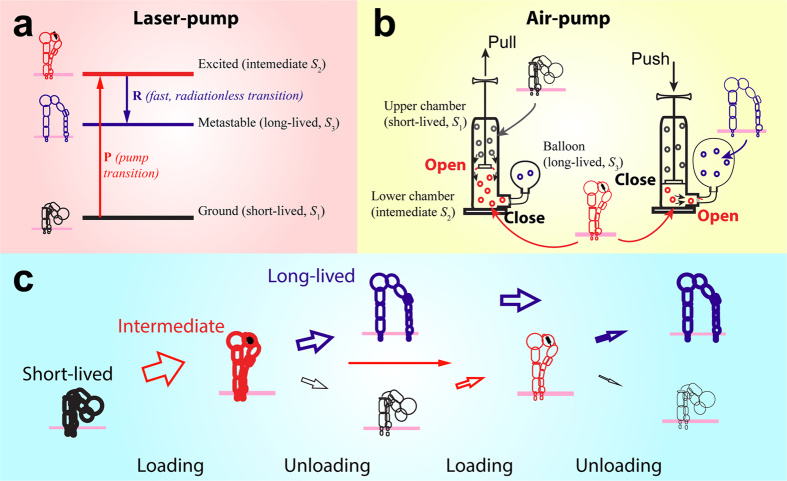
Integrin conformational changes and laser pump/air pump-balloon analogies. (**a**) Laser pump analogy. (**b**) Air pump-balloon analogy. (**c**) Force-induced integrin conformational changes. Correspondence between components of the two systems is indicated. Arrows indicate transitions of integrin between conformations, with the arrow size depicting the magnitude of the transition rate. Darkness of the integrin cartoons indicates the occupancy of each conformation. Long-lived state accumulates a fraction of occupancy over multiple force cycles.

To better understand the three-state model, we used the laser-pump (Fig. 7a) and air-pump (Fig. 7b) systems as metaphors to illustrate how it works. Atoms in a laser-pump and air in an air-pump are analogous to receptor–ligand bonds, all of which have three states. The operations of both pumps are governed by similar master equations to those of our three-state model. The state transitions behave similarly under force or electric current. For instance, atoms in the laser-pump reside in the ground, excited, and metastable states. Electric current, analogous to mechanical force, inputs an energy ∆*E*, which lowers the free energy barrier from the ground state to the excited states. Therefore, electric current increases atom excitation rate *k*_12_ from the ground state to the excited state by a factor of 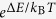, just like mechanical force increases bond transition rate from the short-lived state to the intermediate state by a factor of 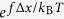. Stopping the electric current resumes the transition rate, and allows the excited atoms to rapidly decay to the metastable state, just like the unloading force allows bonds to rapidly transition from the intermediate state to the long-lived state. Therefore, a stronger electric current or multiple electric current cycles can boost more atoms to the excited state and eventually more atoms in the metastable state. Similarly, force inputs mechanical energy to the air-pump system and the one-way valves guide the air flow from the upper chamber to the lower chamber and to the air balloon. Pulling harder or repeated pulling-pushing cycles input more energy, thus pumping more air to the lower chamber, and eventually to the air balloon.

### Potential relation between integrin states and conformations

Electron microscopic studies reveal the existence of multiple stable conformations for several integrin ectodomains, e.g., bent ectodomain with a closed headpiece and connected legs, extended ectodomain with a closed headpiece and joined legs, and extended ectodomain with an open headpiece and separate legs (Fig. 7c)^40^,^41^,^42^,^43^. Integrins in the bent conformation assume a resting state with a low affinity for ligands^40^. Extended integrins with a closed headpiece and joined legs assume an intermediate state, which is predicted to have an intermediate affinity for ligands^41^,^42^. Extended integrins with an open headpiece and separate legs assume an activated state with a high affinity for ligands^40^,^44^,^45^. It is therefore of interest to relate the force-regulated transitions among the three states in our model to the force-regulated changes in integrin conformations. Three major domain movements are involved in the conformational change from a bent conformation with a closed headpiece and connected legs to an extended conformation with an open headpiece and separate legs: integrin extension, headpiece opening and hybrid domain swing-out, and separation of the legs. The precise order of these movements in integrin conformational changes is still speculative. Xiao *et al*. proposed several conformational transition pathways^41^ induced by different stimulations. Inside-out signaling is predicted to lead to leg separation first. Ligand binding is predicted to stimulate the head piece opening first (outside-in signaling). Additional evidence has been found supporting the inside-out and outside-in activation mechanism^46^,^47^,^48^,^49^,^50^. However, it less clear what biochemical signal would lead to integrin extension. Steered molecular dynamics (SMD) simulations suggest that mechanical force may induce hybrid domain swing-out and integrin extension^51^,^52^. This force-induced extension has been observed in single-molecule experiments^34^,^35^. Crystallographic^40^,^41^ and SMD studies have suggested a downward movement of α7 helices in the αA and/or βA domains. This pistol-like movement has been proposed to relay hybrid domain swing-out to conformational changes at the ligand binding site to result in the high affinity state^41^,^53^. To this end, we propose a conceptual model of integrin conformational changes induced by dynamic forces (Fig. 7c). In this mechanical model, force exerted via an engaged ligand tends to extend the integrin from the bend to extended conformation. Loading promotes the integrin to transition from the short-lived state to the intermediate state, which assumes an extended conformation. Unloading tends to relax the integrin headpiece and allow the downward pistol-like movement of the α7 helices in the αA and/or βA domains to induce the swing-out of the hybrid domain and the separation of the legs. In other words, unloading promotes integrin to transition from the intermediate state to the long-lived state. Increasing the amplitude or number of loading-unloading cycles increases the total flux through the intermediate state, relaying more bond occupancies from the short-lived state to the long-lived state cumulatively, thereby prolonging bond lifetime (Fig. 7c).

### Comparison to two-state model

Chen *et al*. explored the ability of a two-state model to account for the CMR effect^29^. In their model, both time elapses of *T*_0_, starting from the initial time of force application, and *T*_1_, starting from the first instant of force clamping, were simulated. Thus, *T*_0_ includes, but *T*_1_ excludes, the short-lived bonds dissociated during loading-unloading cycles prior to when the experimental bond lifetime measurement begins. The authors were able to generate single-cycled CMR using the same physical mechanism as catch bonds. However, they found that only *T*_1_, but not *T*_0_, exhibited both single- and multi-cycled CMR effects. The apparent prolongation of *T*_1_ in multi-cycled CMR was due to pre-selection of long-lived bonds by cyclic forces, an experimental artifact rather than a real biophysical phenomenon. We note that the possible effect of cyclic forces biasing measurement was ruled out by specifically designed control experiment (Supplementary Fig. S3 in ref. 26). Theoretically two-state models lack the ability to produce multi-cycled CMR effect in *T*_0_. Nor is it possible to generate multi-cycled CMR effect in *T*_1_ without pre-selection of long-lived bonds. Therefore, the need for a three state model stems from the difficulty for any two-state model to account for the multi-cycled CMR. This theoretical assertion can be derived from the use of the Bell equation^4^ to model bond transition between states, which was assumed by Chen *et al*.^29^ and also in the present work (see justification in Supplemental Information). In our model, the intermediate state cyclically relays the transition of short-lived bonds to the long-lived state, which accumulate over repetitive force cycles (Fig. 4), thus prolonging the bond lifetime. Therefore, our three-state model provides a new biophysical mechanism for strengthening of molecular bonds that is distinct from that for catch bonds, which can account for the more complex CMR data. Future studies will examine whether and how our three-state model may describe other forms of force history-dependent mechanical regulation of molecular bond dissociation.

## Methods

We assume that all bonds initially reside in the short-lived state. The master equation was numerically integrated along different prior force histories to calculate bond lifetimes resulted in the force-clamp phase. Three prior force histories were considered, corresponding three experimental assays: force-clamp, single-cycled CMR, and multi-cycled CMR. In the loading phase, force increased linearly with time, with a loading rate of 1000 pN/s. To mimic the experiments, unloading of single-cycled and multi-cycled CMRs was controlled by a proportional–integral–derivative algorithm:


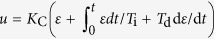


Here *u* is the increment for the current time step, *ε* is the difference between the aim value and the current value. The parameters *K*_C_, *T*_i_, and *T*_d_ are set to be 0.0001, 6 s, and 0 s, respectively.

Since high forces lead to fast transition of bond occupancies between states, the time step during loading-unloading was set to be 0.01 μs. To save computational time, the time step in the low force clamping phase was set to be 1 μs. To avoid pre-matured bond dissociation, we constrained the dissociation during loading and unloading. Computed bond survival distributions and force-lifetime relationships were compared to the experimental data (Fig. 3) by Monte Carlo Least-Squares fitting^32^ to obtain the best-fit model parameters. With best-fit parameters, we further calculated the fraction of bonds dissociated from each state, 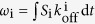, the real-time occupancies, 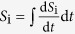, and average lifetime, 
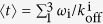
, (*i* = 1, 2, 3).

## Additional Information

**How to cite this article**: Li, Z. *et al*. A model for cyclic mechanical reinforcement. *Sci. Rep.* **6**, 35954; doi: 10.1038/srep35954 (2016).

**Publisher’s note:** Springer Nature remains neutral with regard to jurisdictional claims in published maps and institutional affiliations.

## Supplementary Material

Supplementary Information

## Figures and Tables

**Figure 1:**
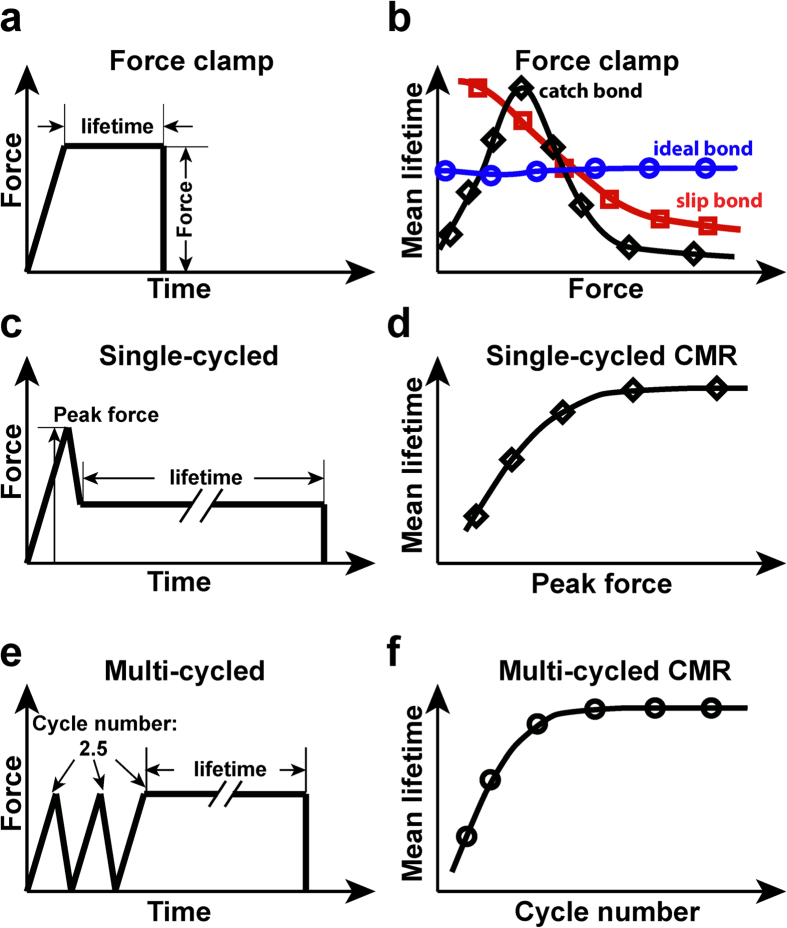
Force-regulation of molecular bond. (**a,c,e)** Representative force-time traces of three different force histories: force clamp (**a**), single-cycled CMR (**c**), and multi-cycled CMR (**e**). (**b**) Representative mean lifetime vs. force plot of three types of force regulation under constant clamping force: catch bond (black symbols and curve), slip bond (red symbols and curve), ideal bond (blue symbols and curve). (**d**) Representative mean lifetime vs. peak force plot of single-cycled CMR. (**f**) Representative mean lifetime vs. cycle number plot of multi-cycled CMR.

## References

[b1] Chan C. E. & Odde D. J. Traction dynamics of filopodia on compliant substrates. Science 322, 1687–1691 (2008). 19074349 10.1126/science.1163595

[b2] Chen C. S. Mechanotransduction–a field pulling together? Journal of cell science 121, 3285–3292 (2008). 18843115 10.1242/jcs.023507

[b3] Gardel M. L. . Traction stress in focal adhesions correlates biphasically with actin retrograde flow speed. The Journal of cell biology 183, 999–1005 (2008). 19075110 10.1083/jcb.200810060 PMC2600750

[b4] Bell G. I. Models for the specific adhesion of cells to cells. Science 200, 618–627 (1978). 347575 10.1126/science.347575

[b5] Liu B., Chen W. & Zhu C. Molecular Force Spectroscopy on Cells. Annual review of physical chemistry 66, 427–451 (2015). 10.1146/annurev-physchem-040214-121742 25580628

[b6] Dembo M., Torney D. C., Saxman K. & Hammer D. The Reaction-Limited Kinetics of Membrane-to-Surface Adhesion and Detachment. Proceedings of the Royal Society of London. Series B, Biological Sciences 234, 55–83, 10.2307/36290 (1988). 2901109

[b7] Rakshit S., Zhang Y., Manibog K., Shafraz O. & Sivasankar S. Ideal, catch, and slip bonds in cadherin adhesion. Proceedings of the National Academy of Sciences 109, 18815–18820 (2012). 10.1073/pnas.1208349109 PMC3503169 23112161

[b8] Marshall B. T. . Direct observation of catch bonds involving cell-adhesion molecules. Nature 423, 190–193 (2003). 12736689 10.1038/nature01605

[b9] Sarangapani K. K. . Low force decelerates L-selectin dissociation from P-selectin glycoprotein ligand-1 and endoglycan. Journal of Biological Chemistry 279, 2291–2298 (2004). 14573602 10.1074/jbc.M310396200

[b10] Guo B. & Guilford W. H. Mechanics of actomyosin bonds in different nucleotide states are tuned to muscle contraction. Proceedings of the National Academy of Sciences 103, 9844–9849 (2006). 10.1073/pnas.0601255103 PMC1502541 16785439

[b11] Kong F., García A. J., Mould A. P., Humphries M. J. & Zhu C. Demonstration of catch bonds between an integrin and its ligand. The Journal of cell biology 185, 1275–1284 (2009). 19564406 10.1083/jcb.200810002 PMC2712956

[b12] Fiore V. F., Ju L., Chen Y., Zhu C. & Barker T. H. Dynamic catch of a Thy-1–α5β1+ syndecan-4 trimolecular complex. Nature communications 5, 4886 (2014). 10.1038/ncomms5886 25216363

[b13] Liu B., Chen W., Evavold B. D. & Zhu C. Accumulation of dynamic catch bonds between TCR and agonist peptide-MHC triggers T cell signaling. Cell 157, 357–368 (2014). 24725404 10.1016/j.cell.2014.02.053 PMC4123688

[b14] Buckley C. D. . The minimal cadherin-catenin complex binds to actin filaments under force. Science 346, 1254211 (2014). 25359979 10.1126/science.1254211 PMC4364042

[b15] Evans E., Leung A., Heinrich V. & Zhu C. Mechanical switching and coupling between two dissociation pathways in a P-selectin adhesion bond. Proceedings of the National Academy of Sciences of the United States of America 101, 11281–11286 (2004). 15277675 10.1073/pnas.0401870101 PMC509195

[b16] Barsegov V. & Thirumalai D. Dynamics of unbinding of cell adhesion molecules: transition from catch to slip bonds. Proceedings of the National Academy of Sciences of the United States of America 102, 1835–1839 (2005). 15701706 10.1073/pnas.0406938102 PMC548539

[b17] Marshall B. T., Sarangapani K. K., Lou J., McEver R. P. & Zhu C. Force history dependence of receptor-ligand dissociation. Biophysical journal 88, 1458–1466 (2005). 15556978 10.1529/biophysj.104.050567 PMC1305147

[b18] Pereverzev Y. V., Prezhdo O. V., Forero M., Sokurenko E. V. & Thomas W. E. The two-pathway model for the catch-slip transition in biological adhesion. Biophysical journal 89, 1446–1454 (2005). 15951391 10.1529/biophysj.105.062158 PMC1366651

[b19] Lou J. . Flow-enhanced adhesion regulated by a selectin interdomain hinge. The Journal of cell biology 174, 1107–1117 (2006). 17000883 10.1083/jcb.200606056 PMC2064400

[b20] Lou J. & Zhu C. A structure-based sliding-rebinding mechanism for catch bonds. Biophysical journal 92, 1471–1485 (2007). 17142266 10.1529/biophysj.106.097048 PMC1796828

[b21] Springer T. A. Structural basis for selectin mechanochemistry. Proceedings of the National Academy of Sciences 106, 91–96 (2009). 10.1073/pnas.0810784105 PMC2629181 19118197

[b22] Waldron T. T. & Springer T. A. Transmission of allostery through the lectin domain in selectin-mediated cell adhesion. Proceedings of the National Academy of Sciences 106, 85–90 (2009). 10.1073/pnas.0810620105 PMC2629216 19118202

[b23] Le Trong I. . Structural basis for mechanical force regulation of the adhesin FimH via finger trap-like β sheet twisting. Cell 141, 645–655 (2010). 20478255 10.1016/j.cell.2010.03.038 PMC2905812

[b24] Chakrabarti S., Hinczewski M. & Thirumalai D. Plasticity of hydrogen bond networks regulates mechanochemistry of cell adhesion complexes. Proceedings of the National Academy of Sciences 111, 9048–9053 (2014). 10.1073/pnas.1405384111 PMC4078813 24927549

[b25] Pereverzev Y. V., Prezhdo O. V., Thomas W. E. & Sokurenko E. V. Distinctive features of the biological catch bond in the jump-ramp force regime predicted by the two-pathway model. Physical Review E 72, 010903 (2005). 10.1103/PhysRevE.72.010903 16089930

[b26] Kong F. . Cyclic mechanical reinforcement of integrin–ligand interactions. Molecular cell 49, 1060–1068 (2013). 23416109 10.1016/j.molcel.2013.01.015 PMC3615084

[b27] Sarangapani K. K. . Regulation of catch bonds by rate of force application. Journal of Biological Chemistry 286, 32749–32761 (2011). 21775439 10.1074/jbc.M111.240044 PMC3173187

[b28] Li D. & Ji B. Predicted rupture force of a single molecular bond becomes rate independent at ultralow loading rates. Physical review letters 112, 078302 (2014). 24579639 10.1103/PhysRevLett.112.078302

[b29] Chen X., Mao Z. & Chen B. Probing time-dependent mechanical behaviors of catch bonds based on two-state models. Scientific reports 5, 1–9 (2015). 10.1038/srep07868 PMC4297987 25598078

[b30] Thomas W. . Catch-bond model derived from allostery explains force-activated bacterial adhesion. Biophysical journal 90, 753–764 (2006). 16272438 10.1529/biophysj.105.066548 PMC1367101

[b31] Chen W., Lou J. & Zhu C. Forcing switch from short-to intermediate-and long-lived states of the αA domain generates LFA-1/ICAM-1 catch bonds. Journal of Biological Chemistry 285, 35967–35978 (2010). 20819952 10.1074/jbc.M110.155770 PMC2975219

[b32] Kirste B. Least-squares fitting of EPR spectra by Monte Carlo methods. Journal of Magnetic Resonance (1969) 73, 213–224 (1987).

[b33] Campbell I. D. & Humphries M. J. Integrin structure, activation, and interactions. Cold Spring Harbor perspectives in biology 3, a004994 (2011). 21421922 10.1101/cshperspect.a004994 PMC3039929

[b34] Chen W., Lou J., Evans E. A. & Zhu C. Observing force-regulated conformational changes and ligand dissociation from a single integrin on cells. The Journal of cell biology 199, 497–512 (2012). 23109670 10.1083/jcb.201201091 PMC3483124

[b35] Chen Y., Lee H., Tong H., Schwartz M. & Zhu C. Force regulated conformational change of integrin α V β 3. Matrix Biology (2016). 10.1016/j.matbio.2016.07.002 PMC5237428 27423389

[b36] Plotnikov S. V., Pasapera A. M., Sabass B. & Waterman C. M. Force fluctuations within focal adhesions mediate ECM-rigidity sensing to guide directed cell migration. Cell 151, 1513–1527 (2012). 23260139 10.1016/j.cell.2012.11.034 PMC3821979

[b37] Das D. K. . Force-dependent transition in the T-cell receptor β-subunit allosterically regulates peptide discrimination and pMHC bond lifetime. Proceedings of the National Academy of Sciences 112, 1517–1522 (2015). 10.1073/pnas.1424829112 PMC4321250 25605925

[b38] Hong J. . Force-Regulated *In Situ* TCR–Peptide-Bound MHC Class II Kinetics Determine Functions of CD4+ T Cells. The Journal of Immunology 195, 3557–3564 (2015). 26336148 10.4049/jimmunol.1501407 PMC4592802

[b39] Liu B. . The cellular environment regulates *in situ* kinetics of T‐cell receptor interaction with peptide major histocompatibility complex. European journal of immunology 45, 2099–2110 (2015). 25944482 10.1002/eji.201445358 PMC5642113

[b40] Takagi J., Petre B. M., Walz T. & Springer T. A. Global conformational rearrangements in integrin extracellular domains in outside-in and inside-out signaling. Cell 110, 599–611 (2002). 12230977 10.1016/s0092-8674(02)00935-2

[b41] Xiao T., Takagi J., Coller B. S., Wang J.-H. & Springer T. A. Structural basis for allostery in integrins and binding to fibrinogen-mimetic therapeutics. Nature 432, 59–67 (2004). 15378069 10.1038/nature02976 PMC4372090

[b42] Luo B.-H., Carman C. V. & Springer T. A. Structural basis of integrin regulation and signaling. Annual review of immunology 25, 619–647 (2007). 10.1146/annurev.immunol.25.022106.141618 PMC1952532 17201681

[b43] Springer T. A. & Dustin M. L. Integrin inside-out signaling and the immunological synapse. Current opinion in cell biology 24, 107–115 (2012). 22129583 10.1016/j.ceb.2011.10.004 PMC3294052

[b44] Mould A. P. . Conformational changes in the integrin βA domain provide a mechanism for signal transduction via hybrid domain movement. Journal of Biological Chemistry 278, 17028–17035 (2003). 12615914 10.1074/jbc.M213139200

[b45] Takagi J., Strokovich K., Springer T. A. & Walz T. Structure of integrin α5β1 in complex with fibronectin. The EMBO journal 22, 4607–4615 (2003). 12970173 10.1093/emboj/cdg445 PMC212714

[b46] Kim C., Ye F., Hu X. & Ginsberg M. H. Talin activates integrins by altering the topology of the β transmembrane domain. The Journal of cell biology 197, 605–611 (2012). 22641344 10.1083/jcb.201112141 PMC3365499

[b47] Nagae M. . Crystal structure of α5β1 integrin ectodomain: Atomic details of the fibronectin receptor. The Journal of cell biology 197, 131–140 (2012). 22451694 10.1083/jcb.201111077 PMC3317794

[b48] Zhu J., Zhu J. & Springer T. A. Complete integrin headpiece opening in eight steps. The Journal of cell biology 201, 1053–1068 (2013). 23798730 10.1083/jcb.201212037 PMC3691460

[b49] Provasi D., Negri A., Coller B. S. & Filizola M. Talin‐driven inside‐out activation mechanism of platelet αIIbβ3 integrin probed by multimicrosecond, all‐atom molecular dynamics simulations. Proteins: Structure, Function, and Bioinformatics 82, 3231–3240 (2014). 10.1002/prot.24540 PMC5156318 24677266

[b50] Xia W. & Springer T. A. Metal ion and ligand binding of integrin α5β1. Proceedings of the National Academy of Sciences 111, 17863–17868 (2014). 10.1073/pnas.1420645111 PMC4273411 25475857

[b51] Puklin-Faucher E., Gao M., Schulten K. & Vogel V. How the headpiece hinge angle is opened: new insights into the dynamics of integrin activation. The Journal of cell biology 175, 349–360 (2006). 17060501 10.1083/jcb.200602071 PMC2064575

[b52] Chen W. . Molecular dynamics simulations of forced unbending of integrin α v β 3. PLoS Comput Biol 7, e1001086 (2011). 21379327 10.1371/journal.pcbi.1001086 PMC3040657

[b53] Xiang X. . Structural basis and kinetics of force-induced conformational changes of an αA domain-containing integrin. PloS one 6, e27946 (2011). 22140490 10.1371/journal.pone.0027946 PMC3225382

