# Supplementary material for: A model for cyclic mechanical reinforcement
Source: Sci Rep. 2016 Oct 27;6:35954. doi: 10.1038/srep35954 (PMC5081513; doi:10.1038/srep35954)
Supplement: Supplementary Information [file srep35954-s1.doc]

**Supplementary information**

**A model for cyclic mechanical reinforcement**

Zhenhai Li, Fang Kong, Cheng Zhu

**SUPPLEMENTARY FIGURES**

**Figure. S1.** Fraction of bonds ruptured from each state vs. peak force or cycle number

**Figure. S2.** Effect of flipping the inner state transition direction

**Figure. S3.** Parameter sensitivity analysis

**Figure. S4.** Effects of loading and unloading rates

**Figure. S5.** Effect of the initial bond distribution on the average bond lifetime.

**Figure. S6.** Effect of removing the no dissociation constraint during loading-unloading on the bond lifetime distribution.

**SUPPLEMENTARY TABLES**

**Table S1:** Summary of kinetic parameters for α_5_β_1_–FN bonds displaying CMR effects

**Table S2:** Summary of kinetic parameters for predicted lifetimes with different off-rates of three states

**Table S3:** Summary of kinetic parameters for TCR–pMHC catch-slip and slip-only bonds

**EXPANDED RESULTS**

**Fractions of bonds dissociated from the three states**

**Effect of flipping the direction from energy well to transition point**

**Effects of loading and unloading rates**

**Difficulty for two-state model to generate multi-cycled CMR effects**

**REFERENCES**

**SUPPLEMENTARY FIGURES**

**
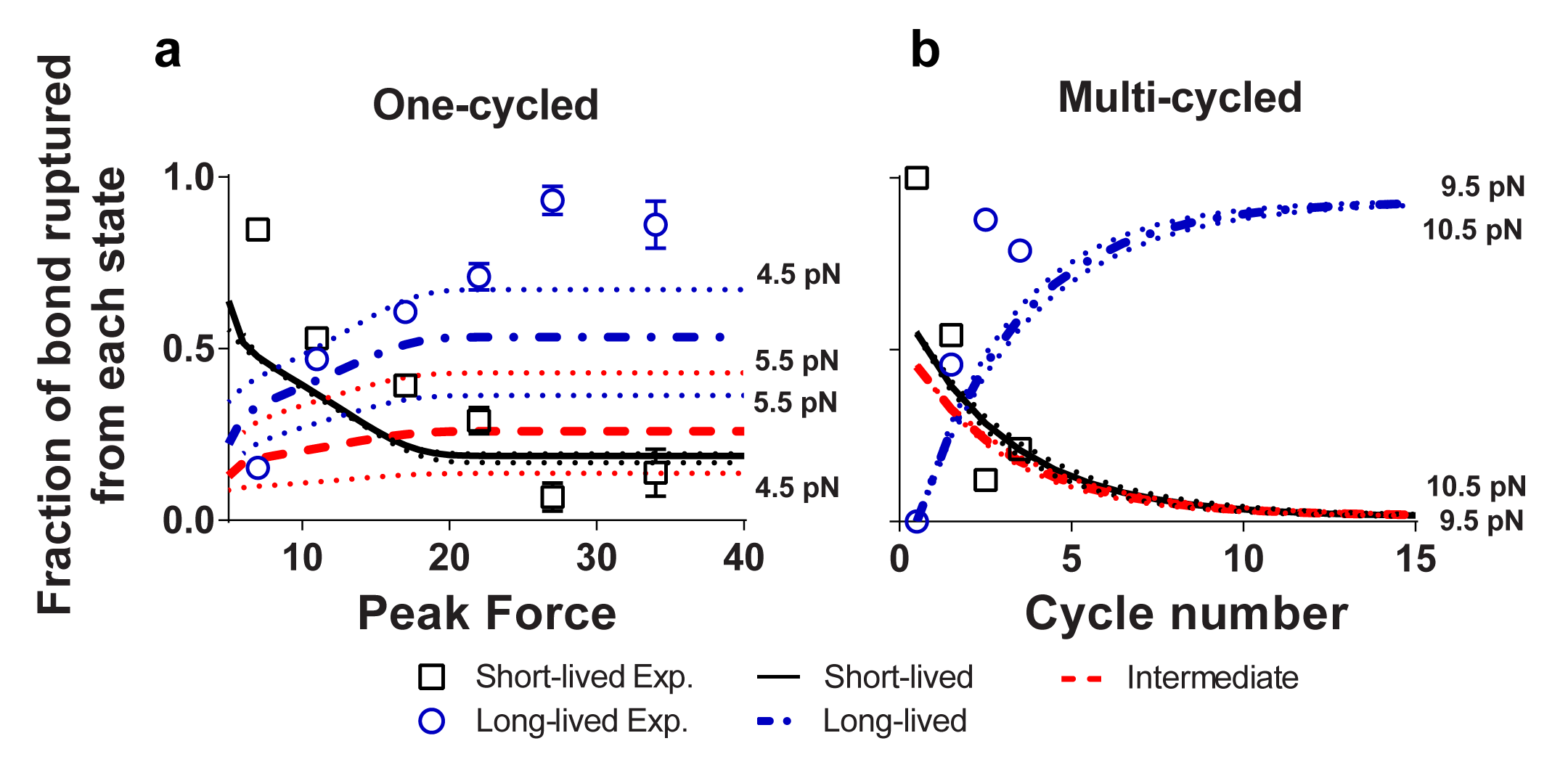
**

**Figure. S1. Fraction of bonds ruptured from each state vs. peak forces (a) or cycle number (b).** The symbols are experimental data from Ref. [1](#_ENREF_1). Dash curves show the model solutions with a range of peak forces followed by clamped forces of 5 pN in single-cycled experiment (a) or with a range of cycle numbers followed by clamped force of 10 pN in multi-cycled experiment (b). The dotted curves indicate solutions with clamped forces ± 0.5 pN about these values. Black, red, and blue colors indicate the short-lived, intermediate, and long-lived states.

**
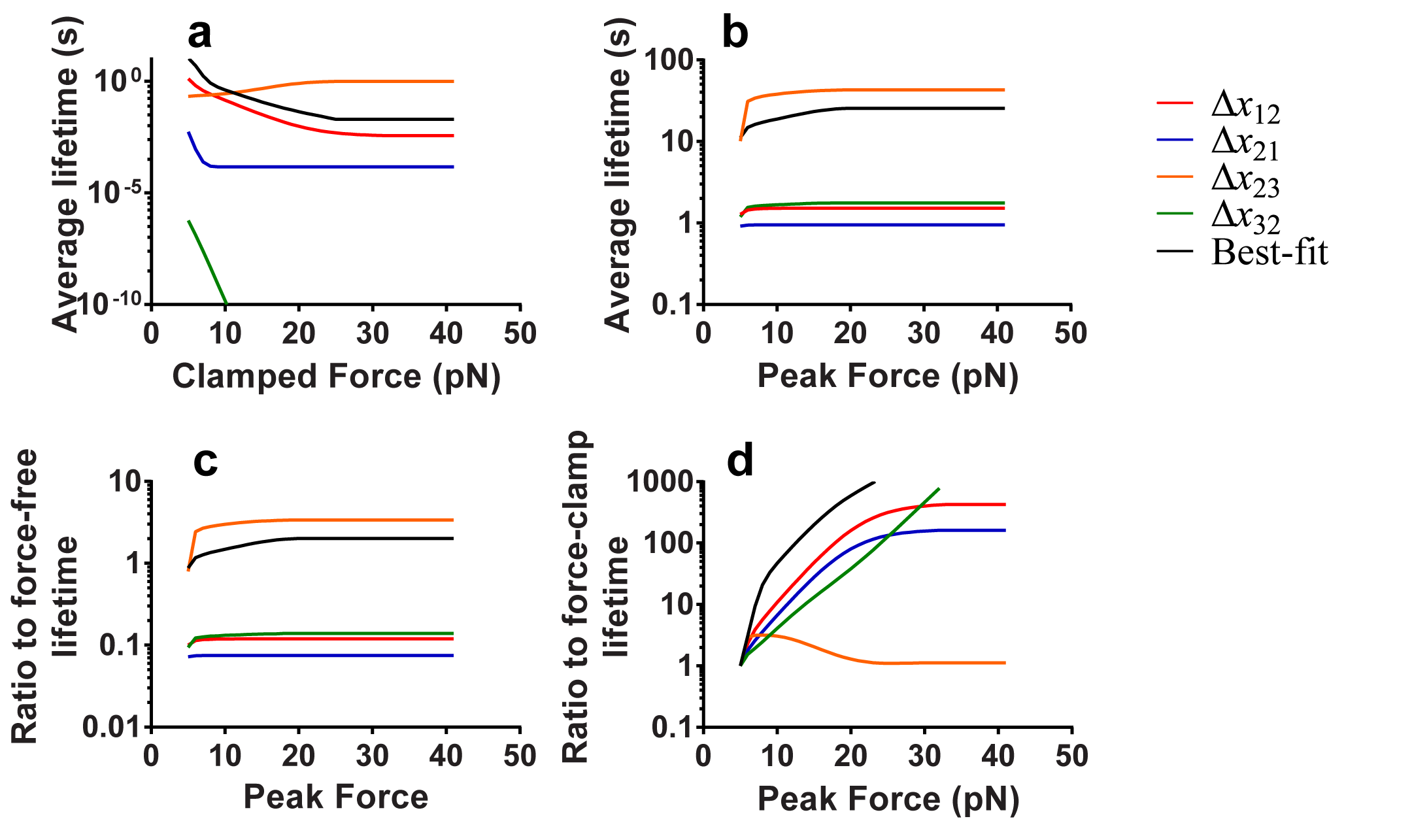
Figure. S2. Effect of flipping the inner state transition direction.** *a, b.* Calculated bond lifetimes post single ramp (a) and post single-cycled CMR (b). *c, d.* The normalized average lifetime post single-cycled loading, by force-free lifetime (c), or by force-clamp lifetime (d). The best fitting for integrin α_5_β_1_-fibronectin (FN) is shown with black curve, and the simulations with flipped ∆*x*_ij_ are indicated by different colors as shown in the figure legend.


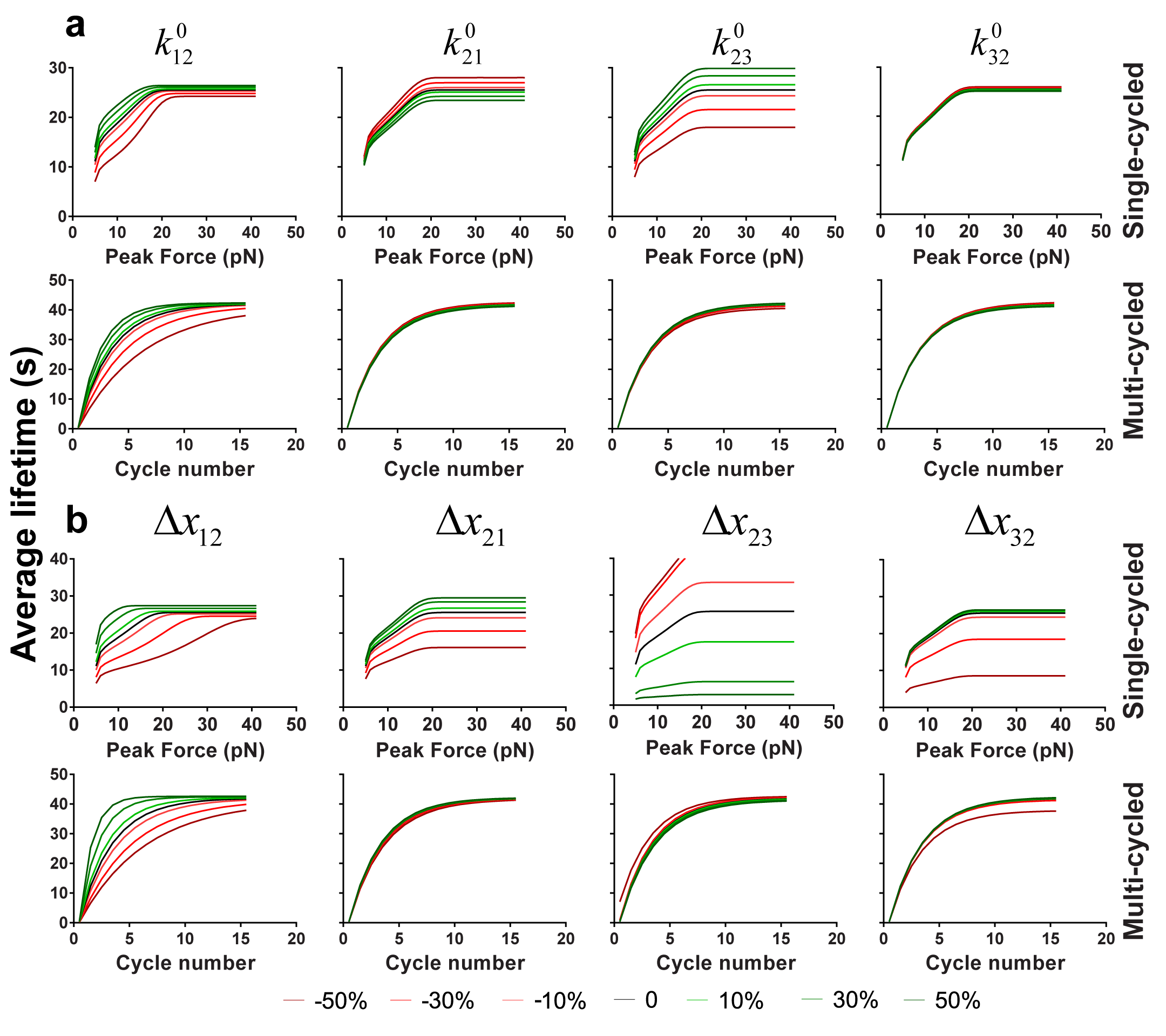


**Figure. S3 Parameter sensitivity analysis.** One-factor-at-a-time approach was used to examine the sensitivity of the average bond lifetime vs. peak force and cycle number curves on the Bell model parameters of the inner-state exchange rates (*Upper row*, for single-cycled CMR; *Lower row*, for multi-cycled CMR). Each parameter was changed from -50% to 50% around the best-fit value while keeping all the other parameters constant as the best-fit value (Supplementary Table S1). *a.* Effects of force-free transition rates$k_{\text{ij}}^{0}$. *b.* Effects of transition distances Δ*x*_ij_. Each column represents the effect of changing a single parameter as indicated.


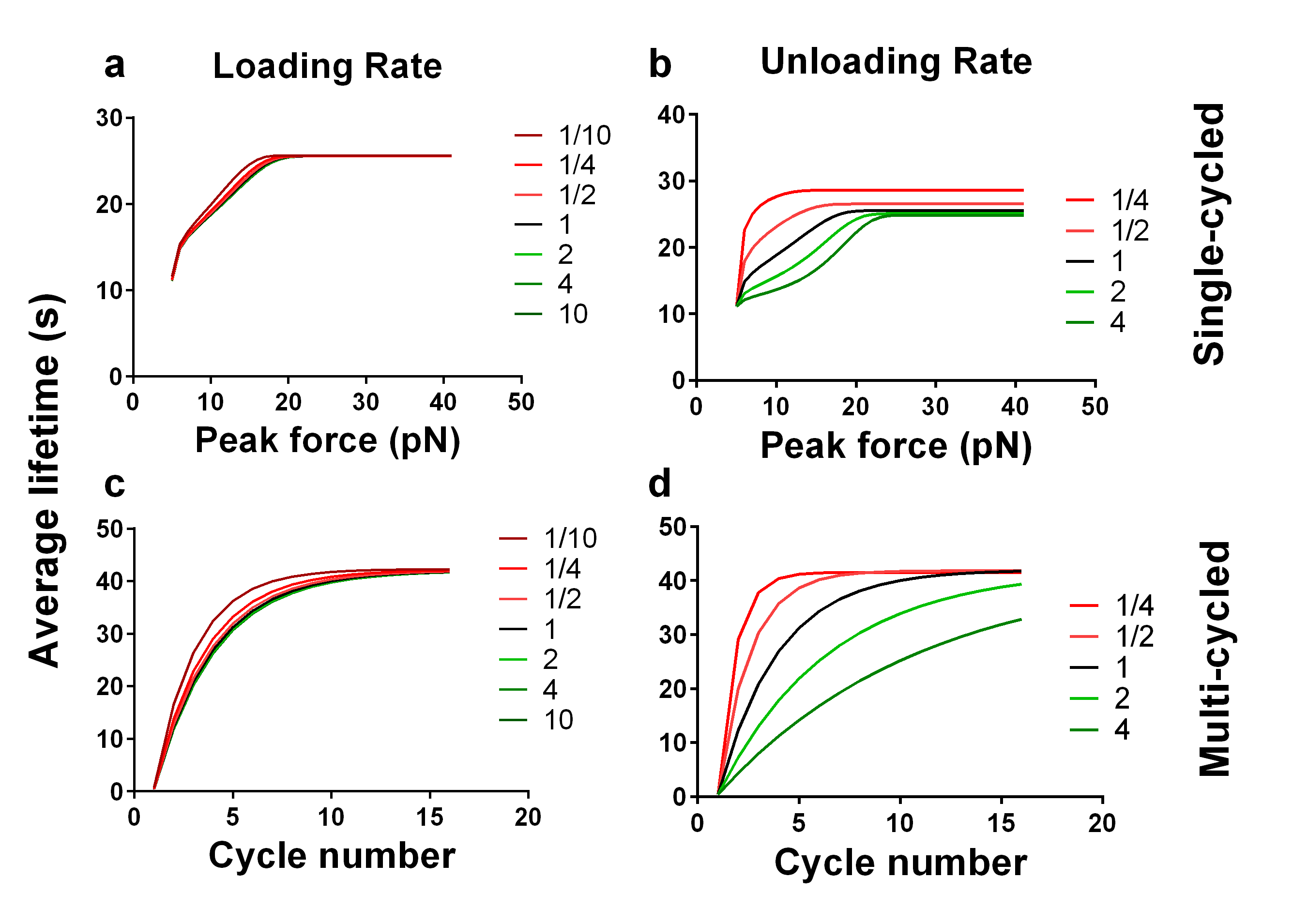


**Figure. S4. Effects of loading and unloading rates on the average bond lifetime.** The loading (a, c) and unloading (b, d) rates were changed with other fitting parameters kept constant to test the effects on bond lifetimes post single-cycled (a, b) and multi-cycled (c, d) CMRs. Loading rate was varied from 1/10 to 10 times of the 1000 pN/s value (denoted as 1 in a&c legends) used in our previous experiments[^1^](#_ENREF_1). The unloading process was controlled by proportional-integral-derivative algorithm, in which the parameter *K*_C_ (see expanded methods below) greatly affected the unloading rate. Therefore in the present testing, *K*_C_ was changed from 1/4 to 4 times of the 0.0001 value (denoted as 1 in b&d legends) used in our previous experiments[^1^](#_ENREF_1)

**
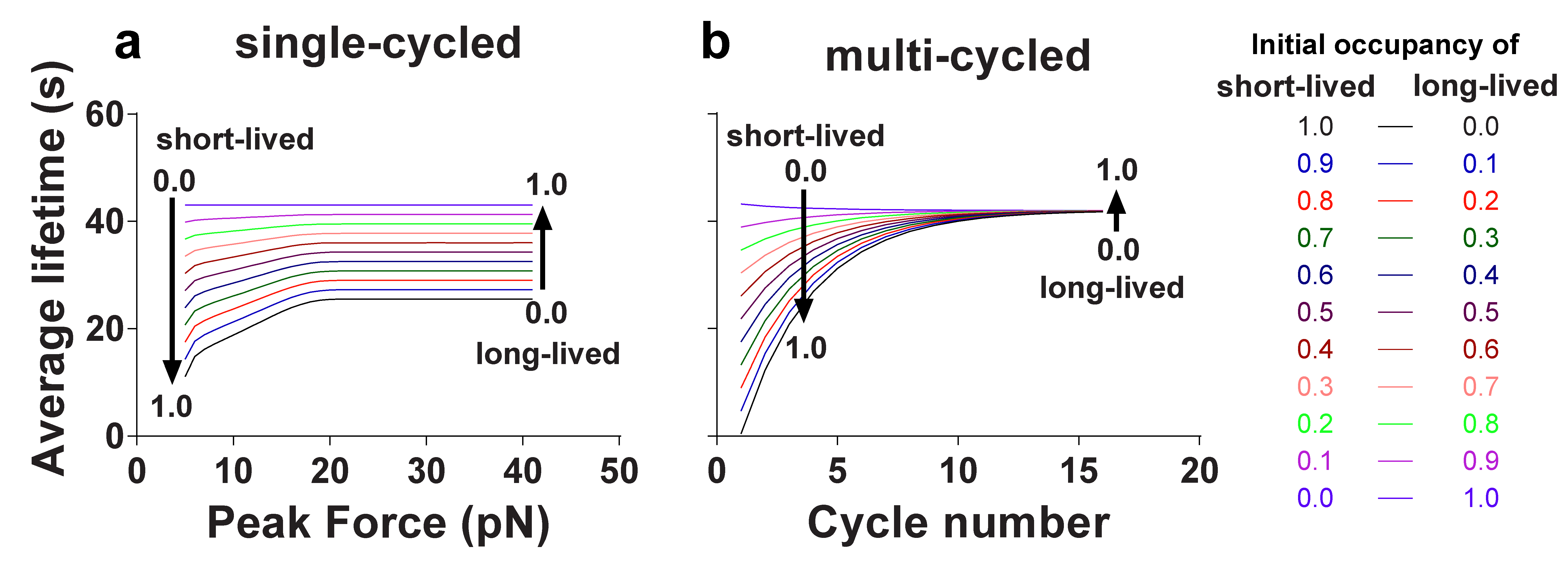
**

**Figure. S5 Effect of the initial bond distribution on the average bond lifetime.** The occupancy of short-lived state is gradually shifted to long-lived state at *t* = 0 with all other parameters kept constant as the best-fit value (Supplementary Table S1) to test the effect of the initial bond distribution on the average bond lifetime in single-cycled (a) and multi-cycled (b) CMR. The initial short-lived state occupancy is reduced from 1 to 0. Correspondingly, the initial long-lived state occupancy is increased from 0 to 1.

**
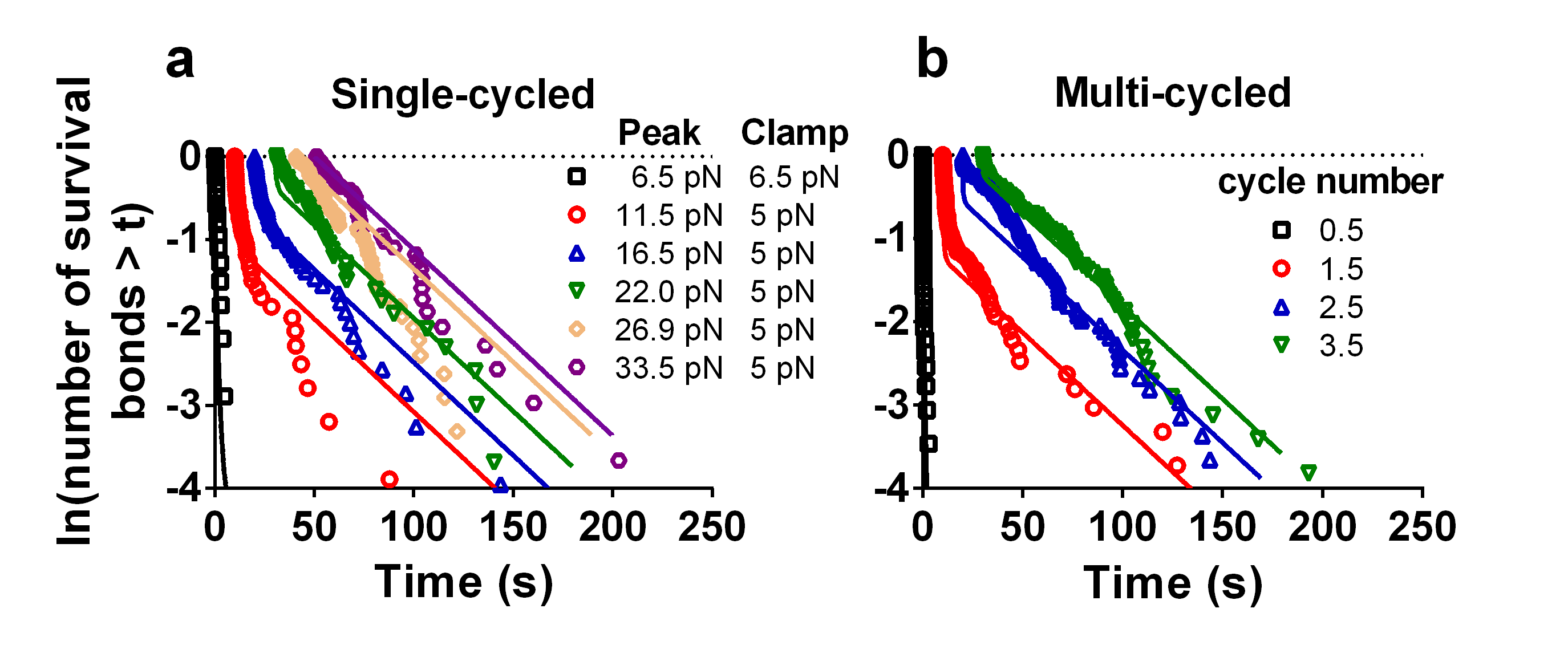
**

**Figure. S6. Effect of removing the no dissociation constraint during loading-unloading on bond lifetime distribution.** The calculated (curves) bond lifetime distributions were fit to those measured (symbols) from single-cycled (a) and multi-cycled (b) CMR experiments after removal of the constraint used in Fig. 3c&d to allow bond dissociation during loading-unloading. The chi squares of single-cycled and multi-cycled fitting are 73.8 and 183.3 respectively. Most of the parameters used to fit Fig. 3c&d were kept the same, except Δ*x*_12_ was changed to 0.96 nm.

**SUPPLEMENTARY TABLES**

**Table 1. Summary of kinetic parameters for α_5_β_1_–FN bonds displaying CMR effects**.

|  | Internal transfer parameters | | | | Dissociation parameters | | |
| --- | --- | --- | --- | --- | --- | --- | --- |
| State | 1→2 | 2→1 | 2→3 | 3→2 | 1 | 2 | 3 |
| *k*^0^ (s^-1^) | 0.22 | 6.13 | 1485 | 8.44 | 0.31 | 0.11 | 0.021 |
| Δ*x* (nm) | 1.35 | -2.27 | -6.28 | -6.87 | 1.31 | 1.08 | 0.03 |

Best-fit parameters of the three-state model to the CMR data from Ref. [1](#_ENREF_1).

**Table 2. Summary of kinetic parameters for predicted lifetimes with different off-rates of three states**.

|  |  | Internal transfer parameters | | | | Dissociation parameters | | | |
| --- | --- | --- | --- | --- | --- | --- | --- | --- | --- |
|  | State | 1→2 | 2→1 | 2→3 | 3→2 | | 1 | 2 | 3 |
| $k_{\text{off}}^{3}<k_{\text{off}}^{1}<k_{\text{off}}^{2}$ | *k*^0^ (s^-1^) | 0.005 | 6.13 | 85 | 8.44 | | 0.05 | 1.11 | 0.01 |
|  | Δ*x* (nm) | 2.86 | -2.3 | -4.1 | -6.8 | | 0.31 | 0.3 | 0.07 |
| $k_{\text{off}}^{1}<k_{\text{off}}^{2}<k_{\text{off}}^{3}$ | *k*^0^ (s^-1^) | 0.005 | 6.13 | 85 | 8.44 | | 0.05 | 1.11 | 10.5 |
|  | Δ*x* (nm) | 2.86 | -2.3 | -4.1 | -6.8 | | 0.31 | 0.3 | 0.37 |
| $k_{\text{off}}^{3}<k_{\text{off}}^{2}<k_{\text{off}}^{1}$ | *k*^0^ (s^-1^) | 0.005 | 6.13 | 85 | 8.44 | | 1.11 | 0.05 | 0.01 |
|  | Δ*x* (nm) | 2.86 | -2.3 | -4.1 | -6.8 | | 0.31 | 0.3 | 0.07 |
| $k_{\text{off}}^{2}<k_{\text{off}}^{1}<k_{\text{off}}^{3}$ | *k*^0^ (s^-1^) | 0.005 | 6.13 | 85 | 8.44 | | 1.11 | 0.05 | 10.5 |
|  | Δ*x* (nm) | 2.86 | -2.3 | -4.1 | -6.8 | | 0.31 | 0.3 | 0.38 |

**Table 3. Summary of kinetic parameters for TCR–pMHC catch-slip and slip-only bonds.**

| Peptide |  | Internal transfer parameters | | | | Dissociation parameters | | |
| --- | --- | --- | --- | --- | --- | --- | --- | --- |
|  | State | 1→2 | 2→1 | 2→3 | 3→2 | 1 | 2 | 3 |
| OVA | *k*^0^ (s^-1^) | 0.12 | 2.99 | 0.50 | 0.02 | 8.49 | 0.52 | 0.00 |
|  | Δ*x* (nm) | 2.75 | -20.9 | -1.59 | -0.25 | 0.83 | 0.41 | 0.00 |
| A2 | *k*^0^ (s^-1^) | 0.09 | 6.57 | 1.21 | 0.06 | 42.4 | 8.24 | 0.00 |
|  | Δ*x* (nm) | 2.15 | -4.59 | -0.38 | -0.65 | 0.42 | 0.66 | 0.00 |
| G4 | *k*^0^ (s^-1^) | 0.03 | 2.08 | 0.19 | 0.01 | 3.59 | 1.69 | 0.10 |
|  | Δ*x* (nm) | 7.64 | -14.5 | -5.89 | -0.13 | 0.35 | 1.35 | 0.06 |
| R4 | *k*^0^ (s^-1^) | 0.003 | 1.03 | 2.67 | 0.00 | 2.59 | 1.48 | 0.00 |
|  | Δ*x* (nm) | 2.82 | -7.24 | -8.42 | -0.00 | 0.85 | 0.58 | 0.00 |
| E1 | *k*^0^ (s^-1^) | 0.23 | 0.57 | 0.01 | 0.06 | 2.06 | 1.72 | 0.00 |
|  | Δ*x* (nm) | 5.23 | -3.98 | -0.00 | -0.71 | 0.20 | 1.37 | 0.00 |

Best-fit parameters of the three-state model to the data from Ref. [2](#_ENREF_2) for OT1 TCR bonds with the indicated peptides presented by MHC.

**EXPANDED RESULTS**

**Fractions of bonds dissociated from the three states**

Bonds subjected to different dynamic force waveforms are driven to occupy three states differentially to dissociate along three pathways to result different lifetimes. This can be observed from the bond survival distributions. The steep single exponential decays of the data from the 6.5 pN (Fig. 3c) and 10 pN (Fig. 3d) force-clamp experiments indicate that these bonds dissociated from a single, short-lived state. At 16.5 pN peak force in the case of single-cycled CMR (Fig. 3c) or 1.5 cycles in the case of multi-cycled CMR (Fig. 3d), another subpopulation with a slower decay rate appears. Further increase in the peak force or cycle number increases the slow decaying subpopulation at the expense of the fast decaying subpopulation, but does not alter the two dissociation rates, revealing a progressive shift of bond dissociation from the short- to long-lived states, until the distribution becomes single slow decaying exponential again when most bonds dissociate from the long-lived state. Neglecting the intermediate state, and fitting these data[^1^](#_ENREF_1) with a two-state model provided the rough estimates of experimental fractions of bonds dissociated from the short-lived and long-lived states (Fig. S1).

To compare with experiment, we calculated the fractions, $\omega_{\text{i}}$, of bonds dissociated from the *i*th states using the model solution $\int_{t>t_{\text{C}}} S_{\text{i}}k_{\text{off}}^{\text{i}}\text{d}t$ (*i* = 1-3), where $t_{\text{C}}$ is the starting time of clamping. The calculation used the best-fit parameters evaluated from fitting of the model to the bond lifetime data (Supplementary Table S1). For the single-cycled CMR at 5-pN peak force, the fractions of bonds dissociated from the short-lived state and the long-lived state were ~60 and ~20%, respectively. As the peak force increased, the fraction of bonds dissociated from the long-lived state rapidly increased at the expense of the fraction of bonds dissociated from the short-lived state, which rapidly decreased, until both approached their respective plateaus of ~60 and 20% at 20-pN peak force (Fig. S1a). Similarly, for the multi-cycled CMR at 0.5 cycle (i.e., a single loading to the clamped force without unloading), the fractions of bonds dissociated from the short-lived state and the long-lived state were 60 and 0%, respectively. As the cycle number increased, the fraction of bonds dissociated from the long-lived state rapidly increased at the expense of the fraction of bonds dissociated from the short-lived state, which rapidly decreased, until both reached their respective plateaus of 90 and 0% at 9.5 cycles (Fig. S1b). The predicted intermediate state fractions were generally less than 15%, consistent with the experimental distributions (Fig. 3c&d). These favorable comparisons further support the validity of our model and the robustness of the best-fit parameters. Similar to lifetime measurement, the fraction of bonds dissociated from each state has differential sensitivities to clamped force in multi-cycled vs. single-cycled CMR. ± 0.5 pN deviations in the clamped force lead to ~10% changes in intermediate and long-lived bond fractions in single-cycled CMR, but not in multi-cycled CMR.

**Effect of flipping the direction from energy well to transition point**

The sign of Δ*x*_ij_ corresponds to the direction from the bottom of the *i*th energy well to the point of transition to the *j*th energy well (Fig. 2b&c), which determines whether the *i* → *j* state transition will be accelerated or decelerated by force comparing to the force-free transition rate $k_{\text{ij}}^{0}$, and thus is critical to the CMR effect. To test how these directions affect CMR, we integrated the master equation along single-cycled force loading histories and calculated the average lifetimes with the direction of one Δ*x*_ij_ flipped, but the others kept the same. The calculated average lifetimes with force-clamp and single-cycled force histories were plotted in Fig. S2a&b. Beside the best-fitting curve, flipping Δ*x*_23_ prolongs lifetime with single-cycled loading history (Fig. S2b). In other cases, significant prolongation of average lifetime is not observed. In order to better compare the CMR effects, we normalized the average lifetimes under single-cycled loading either by force-free lifetime, or by force-clamp lifetime. Normalizing with force-free lifetime shows if the cyclic forces strengthen the bonds or not. Normalizing with force-clamp lifetime shows whether bond strengthening is due to the catch bond mechanism or CMR. Although flipping Δ*x*_23_ significantly prolongs bond lifetime under single-cycled force history (Fig. S2b&c), the prolongation is mainly due to a catch bond mechanism (Fig. S2a&d).

**Effects of loading and unloading rates**

We applied the model to predict the behavior of bonds subjected to different loading histories. We simulated a set of force histories with a range of loading or unloading rates. Keeping unloading rate constant and varying loading rates from 1/10 to 10 times of that used experimentally, we found negligible impact on average bond lifetimes when the peak force (for single-cycled CMR) and cycle number (for multi-cycled CMR) are high, but those numbers slightly change at low peak forces and cycle numbers. Keeping the loading rate constant and varying the unloading rates from ~1/4 to ~4 times from the experimental value, we found similar changes in the average bond lifetimes at low peak force (for single-cycled CMR) and cycle number (for multi-cycled CMR) (Fig. S4).

**Difficulty for two-state model to generate multi-cycled CMR effects**

Any two-state model can be treated as a reduction of our three-state model in which both the inner state transitions and dissociations obey first-order kinetics (all the parameters used here have the same definition as in main text):

$\frac{\text{d}S_{1}}{\text{d}t}=-S_{1}k_{\text{off}}^{1}-S_{1}k_{12}+S_{2}k_{21}$ (S1)

$\frac{\text{d}S_{2}}{\text{d}t}=-S_{2}k_{\text{off}}^{2}-S_{2}k_{21}+S_{1}k_{12}$ (S2)

In two-state catch bond model, force prolongs lifetime by driving bond occupancies from the short-lived state (state 1) to the long-lived state (state 2), resulting more bonds dissociated from the long-lived state and less bonds dissociated from the short-lived state. To further prolong the bond lifetime by cyclic forces, the fraction of bonds dissociated from the long-lived state needs to be even higher than that under force clamp. Since bonds dissociate eventually regardless of their states, both *S*_1_ and *S*_2_ vanish at *t* → ∞. It follows the integration of Eq. (S1&S2) that the fraction of bonds those dissociate from *i*th state, *ω*_i_, is equal to the initial occupancy plus an integral of inner transition flux over time:

$\omega_{\text{i}}=\int_{0}^{\infty} S_{\text{i}}k_{\text{off}}^{\text{i}}\text{d}t=S_{\text{i}}|_{\text{t}=0}+\int_{0}^{\infty} (S_{\text{j}}k_{\text{ji}}\text{-}S_{\text{i}}k_{\text{ij}}\text{)d}t=S_{\text{i}}|_{\text{t}=0}+\int_{0}^{\infty} S_{\text{j}}k_{\text{ij}}(k_{\text{ji}}\text{/}k_{\text{ij}}\text{-}S_{\text{i}}\text{/}S_{\text{j}}\text{)d}t$ (S2)

where *i* and *j* = 1 or 2, *i* ≠ *j*. The initial state occupancies *S*_i_|*_t_*_=0_ are assumed the same in order for us to compare the abilities of force clamp, single-cycled, and multi-cycled loading histories to prolong bond lifetime.

To elicit catch bonds using force-clamped experiment, it requires *k*_12_/*k*_21_ to increase with increasing force such that bonds will transition from *S*_1_ to *S*_2_. To generate multi-cycled CMR effects requires *ω*_2_ to increase with the cycle. Comparing force clamp and multi-cycled loading, when *t* < *t*_C_, *f*_force clamp_ ≥ *f*_multi-cycled_, where *t*_C_ is the beginning of clamping in multi-cycled experiment. Since *k*_12_/*k*_21_ increases with force increase, *k*_12_/*k*_21_ in multi-cycled loading is smaller than that in force clamp. Thus the accumulation over time of net flux from short-lived to long-lived state of multi-cycled loading is smaller than force clamp, which eventually leads to less bonds transitioned to long-lived state. In contrast, comparing force clamp and single-cycled loading, when *t* < *t*_C_, *f*_force clamp_ $\leq$ *f*_single-cycled_. Thus single-cycled loading leads to greater accumulation of transition net flux to long-lived state. As a result more bonds would transition to long-lived state at *t*_C_. Therefore with a two-state model, the lifetime could be prolonged by single-cycled force, but not by multi-cycled loading-unloading unless with selection of the long-lived bond during loading-unloading as shown in Ref [3](#_ENREF_3).

**REFERENCES**

1 Kong, F. *et al.* Cyclic mechanical reinforcement of integrin–ligand interactions. *Molecular cell* **49**, 1060-1068 (2013).

2 Liu, B., Chen, W., Evavold, B. D. & Zhu, C. Accumulation of dynamic catch bonds between TCR and agonist peptide-MHC triggers T cell signaling. *Cell* **157**, 357-368 (2014).

3 Chen, X., Mao, Z. & Chen, B. Probing time-dependent mechanical behaviors of catch bonds based on two-state models. *Scientific reports* **5**, 1-9 (2015).
